# Growth status and menarcheal age among adolescent school girls in Wannune, Benue State, Nigeria

**DOI:** 10.1186/1471-2431-10-60

**Published:** 2010-08-19

**Authors:** Daniel T Goon, Abel L Toriola, Jonathan Uever, Sarah Wuam, Olutoyin M Toriola

**Affiliations:** 1 Centre for Biokinetics, Recreation and Sport Science, University of Venda, Thohoyandou, South Africa; 2 Department of Sports, Rehabilitation and Dental Sciences, Tshwane University of Technology, Pretoria, South Africa; 3 Department of Human Kinetics and Health Education, Benue State University, Makurdi, Nigeria; 4 Department of Physical and Health Education, College of Education, Katsina-Ala, Nigeria; 5 Department of Primary Education (Physical Education Unit), University of Swaziland, Kwaluseni, Swaziland

## Abstract

**Background:**

Menarcheal age is a sensitive indicator of environmental conditions during childhood. The aim of study is to determine the age at menarche and growth status in adolescents in a rural area of Tarka, Wannune, Nigeria.

**Methods:**

Data on 722 female students (aged 12-18 years) were collected in February 2009. Height and weight were measured. Body mass index (BMI; kg m^-2^) was used as an index of relative weight.

**Results:**

Mean and median menarcheal age calculated by probit analysis were 13.02 (SD 3.0) (95% CI: 13.02-13.07), and age 13.00 (SD 2.8) (95% CI: 12.98-13.04), respectively. Girls who reach menarche are significantly heavier and taller with higher BMIs than those of their pre-menarcheal peers.

**Conclusion:**

The age of menarche is probably still declining in Nigeria. Although BMI is an important factor in the onset of menstruation, some other unmeasured environmental variables may be implicated in this population.

## Background

Menarcheal age is the most widely used indicator of sexual maturation and is known to be influenced by genetic factors, environmental conditions, body stature, family size, body mass index (BMI), socioeconomic status and level of education [1-3]. It is the most accurately recalled indicator of puberty among girls [4]. It varies between individuals and populations [5]. Female anthropometry that reveals body composition has shown strong influence on their reproductive characteristics marked by the menarcheal age [6]. An early menarcheal age is associated with increased risk for breast cancer [7], obesity [8], endometrial cancer [9] and uterine leiomyomata [10]. Also, several studies have reported that age at menarche may relate to subsequent reproductive performance, such as age at first intercourse, age at first pregnancy and risk of subsequent miscarriage [11].

The adolescent age group (10-19 years of age) constitutes about 23% of Nigeria's population [12] and this proportion is projected to be stable in the foreseeable future. There is strong evidence to confirm that these adolescents are sexually active [13]. Tarka is a relatively new local government council in Nigeria, which is disadvantaged in terms of health and socio-economic indicators. Reliable information about sexuality, contraception and sexually transmitted infections in Tarka Local Government Area (LGA) is lacking. For the majority of people in the local government, sexuality and menstruation are still social taboos.

Several studies have reported age at menarche to have declined in developed countries [14-16] and this decline has also been noted in developing countries [17,18]. Generally, these declines have been associated with improvements in nutritional status and general health along with many environmental factors. The downward trend seems to have halted in some countries [19]. The age of girls who start menstruating is an important factor in health planning, especially relating to the provision of sanitary facilities, health information concerning menstruating and contraception in primary and secondary schools, and the establishment of adolescent health centres [20].

The relationship between growth and menarche remains debatable. Stark et al [21] content in their study that in affluent individuals nutrition is relatively unimportant. Other studies have indicated that girls who attain menarche are significantly heavier and taller with higher BMIs than those of their pre-menarcheal peers [22,23].

Age at menarche has been reported in several parts of the world [14-16], including eastern, western and northern Nigeria [24-35]. Most of these studies were based on urban samples with limited information available on the menarcheal age of Nigerian girls living in rural communities. Data on menarcheal age may be used to improve health promotion services for girls in rural areas which are often neglected in terms of socio-economic development. The aim of this study was to examine the relationship between current age at menarche and growth status in a cross-section of adolescent girls in Wannune, Tarka LGA, Nigeria.

## Methods

### Sample

This anthropometric survey involved 722 students with an average age of 16.19 years (range: 12-18 years old), attending five government-approved secondary schools in Tarka, Nigeria. Tarka LGA is one of the 23 local government councils in Benue State, Nigeria. With an estimated 134, 123 inhabitants [12], its administrative headquarter is based in Wannune. This relatively new LGA of Benue State is situated in a rural setting, and the major occupation of the people is farming. Others are either employed in white-collar jobs or are involved in private businesses. There were six secondary schools in Wannune as at the time of this study. Most children from all the districts come to live and attend school in the area. Therefore, children who attend schools which participated in this study come from all the districts. This indicates that participants of the study represent a cross-section of children in the area. Participants were selected by simple randomization technique. Parents and schools authorities approved the study protocol before data collection. The Area Education Office of Tarka LGA gave ethical approval for the study. The survey was carried out in February 2009.

### Anthropometric measures

Stature and body mass were determined according to the standard anthropometric methods of the International Society for the Advancement of Kinanthropometry (ISAK) [36]. Stature was measured to the nearest 0.1 cm in bare feet with participants standing upright against a wall-mounted stadiometer. Body mass was measured to the nearest 0.5 kg with participants lightly dressed (underwear and T-shirt) using a portal digital scale (Tanita HD 309, Creative Health Products, MI, USA). BMI was calculated from the ratio of body mass (kg)/stature (m^-2^). Anthropometric measurements were conducted by the author (DTG), a level II ISAK anthropometrist.

### Menarcheal age

Menarcheal status was based on confidential questionnaire responses about date of birth, whether or not the girls had started menstruating, and age of onset in years and months. The different ages were regrouped in classes of 1 year each and were represented by the sign + [22].

### Statistical analysis

Age at menarche was calculated from date of menarche (month and year) and date of birth. Probit analysis was performed to determine the age at menarche for all girls by estimating the age at which 10, 25, 50, 75, and 90% of the girls reached menarche. Analysis was carried out with SPSS software version 17.0. The level of significance was set at p ≤ 0.05.

## Results

A total of 761 girls who were studied, 39 were excluded due to incomplete data. Consequently, 722 questionnaires were analysed. The girls' mean age was 16.19 ± 1.51 years (min. 12, max. 18). Mean BMI was 22.1 ± 2.5 kg m^-2^, with 5.0% of Tarka girls classified as underweight, (BMI < 5th centile of WHO reference) [37]. Figures for overweight and obesity were 9.9%. Distributions of girls according to their age at menarche and those who had not had experienced menarche based on age at the time of the survey is shown in Table 1. A small number of girls started menstruating before the age of 12 years; 5 (16.4%) at age 10 (completed years), and 8 (37.0%) at age 11. A total of 674 (65.2%) respondents experienced menarche. The mean and median ages at menarche among the girls were 13.02 (SD 3.0) (95% CI: 13.02-13.07) and 13.00 (SD 2.8) years (95% CI: 12.98-13.04), respectively. The prevalence of primary amenorrhoea (no menstruation yet at the age of 16 years and more) was 2.6% (six of 320 respondents).

**Table 1:** Number and percentage of menstruating girls (average age for each age band is x + 0.5 years)

		Menstruating girls		Non-menstruating girls
				
Age	Total number of girls	n	%	n
12+	30	13	53.4	17
13+	31	18	58.1	13
14+	67	58	86.6	9
15+	115	112	97.4	3
16+	150	147	97.4	3
17+	170	167	97.4	3
18+	159	159	100.0	
Total	722	674		48

Table 2 compares the anthropometric data according to menarcheal status. The mean values for height, weight and BMI were higher among the post-menarcheal girls compared to their pre-menarcheal contemporaries. The mean weight and BMI were significantly higher for menstruating girls in nearly all the age groups, while there were significant differences in height for ages 13-17 years.

**Table 2:** Growth status of menstruating (post-) and non-menstruating (pre-) girls

	n = 722		Height (cm)		Weight (kg)		**BMI (kg m**^**-2**^**)**	
					
Age	Pre-	Post-	Pre-	Post-	Pre-	Post-	Pre-	Post-
12+	17	13	148.1 ± 6.0	159.0 ± 6.1	45.8 ± 8.2	49.8 ± 4.2*	18.7 ± 2.6	20.2 ± 3.0*
13+	13	18	151.1 ± 4.8	153.7 ± 5.6*	48.0 ± 6.7	51.4 ± 5.6*	21.0 ± 2.8	21.8 ± 2.4
14+	9	58	151. 6 ± 5.2	155.5 ± 5.3*	49.0 ± 4.1	52.2 ± 7.0*	21.3 ± 2.2	21.6 ± 2.6
15+	3	112	146.2 ± 6.7	155. 9 ± 5.9*	42.2 ± 9.8	53.5 ± 6.5*	19.5 ± 2.6	22.0 ± 2.4*
16+	3	147	153.3 ± 4.6	156. 1 ± 5.8*	48.3 ± 5.3	54. 1 ± 7.3*	20.4 ± 2.4	22.1 ± 2.5*
17+	3	167	154.5 ± 5.3	156.9 ± 5.6*	52.4 ± 5.6	55. 6 ± 7.9*	20.1 ± 3.4	22.5 ± 2.9*
18+	0	159		158.1 ± 6.2		55.1 ± 6.1		22.0 ± 2.2
	48	674						

*p < 0.01

Predictors of the onset of menstruation are shown in Table 3. Unadjusted odds ratios indicate, as expected, that older age and higher BMI were positively associated with attainment of menarche. Being only child too, was highly predictive.

**Table 3:** Predictors of onset of menstruation

	Unadjusted OR (95% CI)	Adjusted OR* (95% CI)
Older age	2.5 (1.5-2.2)	2.2 (0.9-2.5)
Higher BMI	3.2 (2.1-4.2)	2.8 (1.8-3.7)
Higher level of father's education	1.3 (0.8-1.6)	not in model
Higher level of mother's education	1.1 (0.5-1.4)	not in model
Only child	2.3 (1.5-3.2)	1.1 (0.7-1.3)

*Logistics regression model: all listed variables included except parental education.

Age and BMI adjusted as continuous variables.

Higher education level defined as completion of at secondary school education to age 18.

## Discussion

Menarcheal age was assessed by *status quo *technique in 722 school girls in Tarka municipality, Nigeria. Among the girls the mean and median ages at menarche were 13.02 and 13.00 years, respectively. The findings contradict the accepted view of delayed menarcheal age in rural communities. The scale (around 1 year) of urban-rural differences lies within the range of variation observed in developing countries [38]. Our data suggest a trend of declining age of menarche in Nigerian girls. Table 4 provides a comparison of mean age at menarche in the present study with those reported in other Nigerian studies. Menarcheal age in this study is slighter lower, but comparable to those reported in previous studies carried out in other parts of Nigeria [24-35], and greater than [24,25,32-34] those studies conducted in various settings. A recent study [35], among school girls in Kaduna, found menarcheal age of 12.81 years (SD 3.1) which is younger than the mean age in our study. Compared to most African and Asian studies, the age at menarche in our study is younger but older than those reported in most European and North American countries, which may reflect different environmental influences. The methods of collecting and analysing data vary in different studies. Thus, attempts at comparing menarcheal ages across studies should be done cautiously.

**Table 4:** Studies on mean age at menarche in different regions

Year	n	Age	Sample	**Age at menarche **^**1**^	Reference
1949	250	8-18	Urban school girls (Lagos)	14.3	[33]
1960-1	335	12-19	Urban school girls (Ibo)	14.07 ± 0.16	[34]
1973-4	2029	10-17	urban school girls (Ibadan)	13.7 ± 0.03	[24]
	328		Rural	14.5 ± 0.09	
1978	1365	10-18	All	13. 54 ± 0.07	[25]
	1216		Urban	13.48 ± 0.09	
	149		Semi-urban	14.05 ± 0.18	
1982-3	2207	10-19	Urban (Enugu)	13.3 ± 1.09	[26]
1985			Mixed (Ilorin)	13.7 ± 1.0	[27]
1990			School girls (Ile-Ife)	13.4 ± 1.4	[28]
	5736	11-18	School girls	13.42 ± 1.51	[29]
	352	13-15	Urban school girls	13.94 ± 1.31	[30]
	859		Urban school girls (Portharcourt)	13.19 ± 1.32	[32]
			Rural school girls (Etche)	14.22 ± 1.47	
2006	358	12-18	School girls (Kaduna)	12.81 ± 1.31	[35]
	900	10-20	Urban school girls (Portharcourt)	13.43 ± 2.19	[31]
2009	722	12-18	Rural school girls (Tarka)	13.02 ± 3.0	[Present study]

^1^Years, median/mean ± SD.

Recent longitudinal studies have consistently indicated that adiposity in early childhood predicts early puberty [39,40]. In our study, BMI was used as a measure of adiposity. The gain in body fat may be one of the key signals, possibly through secretion of the fat-derived protein leptin, for stimulating the hypothalamus to increase secretion of GnRH [41], which in turn stimulates the pituitary-ovarian axis and initiates the pubertal surge. Our findings indicate that girls who reach menarche are significantly heavier and taller with higher BMIs than those of pre-menarcheal status of same age group. This is consistent with findings which reported that BMI is a contributing factor in determining the age of onset of puberty [23,42,43].

The prevalence of amenorrhea in our study was 2.6% (six of 320 respondents who were aged 16 years and older), which falls slightly below the reported prevalence of 3-4% [44,45]. According to Frisch et al [46] in addition to there being a critical weight at which menarche occurs, there is a critically low weight in relation to height at which amenorrhoea develops. A sizeable percentage of the adolescents were underweight (5.0%), therefore the likelihood of amenorrhea is obvious. A study by Ayatollahi et al [47] found that underweight delayed menarche by about 3 months and 3 weeks. Age at menarche is delayed especially in populations with poor nutrition [2,48], and historically, improved nutrition and socioeconomic status has been attributed to causing a decline in the age of menarche [49,50]. The small number of amenorrhea noted in the present study could be ascribed to lesser stress and better nutrition, but this claim needs further investigation. A previous research reported that undernutrition delays menarche and adolescent growth spurt, which normally precedes menarche [51].

## Conclusions

Our data seem to support the theory that BMI is a key factor in the onset of menarche [22,52]. However, as the study was cross-sectional, a cause and effect relationship could not be ascertained. Some of the differences in menarcheal age are likely to be due to other environmental variables which were not included in the study's design such as socio-economic status and general living conditions. Although early menarcheal age may lead to increased body weight, high BMI can in turn cause early menarche and persist in the postmenarcheal period. The findings of the present study support earlier ones and indicate a rate of decline in age at menarche. Also, age at menarche is not delayed in this rural region of Nigeria; rather, it is comparable to urban findings. Improved living conditions may possibly be responsible for the parity.

## Competing interests

The authors declare that they have no competing interests.

## Authors' contributions

DTG was the primary investigator for the study, designed the study, supervised data collection and wrote the paper. ALT advised on data collection, analysed the data and helped write the paper. JU participated in designing the study, analysing the data and writing the paper. SW and OMT advised on the design of the study, the analysis of the findings and writing of the paper. All authors read and approved the final manuscript.

## Pre-publication history

The pre-publication history for this paper can be accessed here:

http://www.biomedcentral.com/1471-2431/10/60/prepub
